# Critical Device Reliability Assessment in Healthcare Services

**DOI:** 10.1155/2023/3136511

**Published:** 2023-02-20

**Authors:** Noorul Husna Abd Rahman, Ayman Khallel Ibrahim, Khairunnisa Hasikin, Nasrul Anuar Abd Razak

**Affiliations:** ^1^Department of Biomedical Engineering, Universiti Malaya, Kuala Lumpur 50603, Malaysia; ^2^Engineering Services Division, Ministry of Health, Putrajaya 62590, Malaysia; ^3^Faculty of Computing and Informatics, Universiti Malaysia Sabah, Sabah 88400, Malaysia

## Abstract

Medical device reliability is the ability of medical devices to endure functioning and is indispensable to ensure service delivery to patients. Preferred Reporting Items for Systematic Review and Meta-Analyses (PRISMA) technique was employed in May 2021 to evaluate existing reporting guidelines on medical device reliability. The systematic searching is conducted in eight different databases, including Web of Science, Science Direct, Scopus, IEEE Explorer, Emerald, MEDLINE Complete, Dimensions, and Springer Link, with 36 articles shortlisted from the year 2010 to May 2021. This study aims to epitomize existing literature on medical device reliability, scrutinize existing literature outcomes, investigate parameters affecting medical device reliability, and determine the scientific research gaps. The result of the systematic review listed three main topics on medical device reliability: risk management, performance prediction using Artificial Intelligence or machine learning, and management system. The medical device reliability assessment challenges are inadequate maintenance cost data, determining significant input parameter selection, difficulties accessing healthcare facilities, and limited age in service. Medical device systems are interconnected and interoperating, which increases complexity in assessing their reliability. To the best of our knowledge, although machine learning has become popular in predicting medical device performance, the existing models are only applicable to selected devices such as infant incubators, syringe pumps, and defibrillators. Despite the importance of medical device reliability assessment, there is no explicit protocol and predictive model to anticipate the situation. The problem worsens with the unavailability of a comprehensive assessment strategy for critical medical devices. Therefore, this study reviews the current state of critical device reliability in healthcare facilities. The present knowledge can be improved by adding new scientific data emphasis on critical medical devices used in healthcare services.

## 1. Introduction

Medical device reliability is defined as the probability of medical devices to perform its intended function for a specified period of time or medical device ability to operate without failure and specifies a future function or performance of a device [1]. Reliability can be defined as dependability and the International Electrotechnical Commission (IEC) defines dependability as “availability performance and its influencing factors: reliability performance, maintainability performance and maintenance support performance” [1]. Previously, mean time between failures (MTBF) was often used to express reliability, whereas mean time to repair (MTTR) expresses maintainability, impacting the device's efficiency. A probabilistic measure of failure-free operation or the ability of equipment to function well without failure during a given time period under certain conditions is another definition for reliability [2]. Therefore, reliability, maintainability, availability, and safety or dependability is the ultimate goal in the maintenance scope. Medical devices should not fail frequently and must be fixed promptly when the failures are detected.

It is reported in many studies that severe injuries and patient death are closely related to faulty medical devices [2]. The World Health Organization (WHO) estimates 50–80% of equipment remains nonfunctional due to lack of maintenance culture, lack of competency, and the tendency to emphasis more on corrective maintenance than preventive maintenance [3]. Deficiency of adequate maintenance of medical devices leads to equipment downtime and reduces device performance, waste cost, and resources [4]. The data recorded in the Malaysian Government Hospitals database proved that hospital operates with massive number of aging medical devices [5]. Equipment failures are one of the challenges, and uptime of the equipment is essential for efficient healthcare delivery in any country. The older technology devices generally demand extra attention due to a lack of service or user manuals and insufficiency of manufacturer recommendations [6]. Sezdi [6] propound user training and maintenance of environmental conditions specified by equipment manufacturers as an action to minimize failures. The event of downtime or equipment failures is predominantly due to improper storage and transportation, initial failure, inappropriate handling (damage during usage), inadequate maintenance, nongenuine spare parts or refurbish spare parts, environmental stress, random failure, and improper handling repair technique and wear out failures [3].

The medical devices maintenance market are growing sophisticatedly driven by the advancement of technology and is forecast to grow at a 9.1% Compound Annual Growth Rate (CAGR) from 2021 to 2026 [7]. According to the WHO, in the USA, approximately 412 US dollars were allocated for medical devices maintenance in 2010 [8]. Besides, the capital expenditure cost for procurement and medical devices system installation is high and contributes almost 40–50% of costs in tertiary hospital setup [9, 10]. An optimal medical maintenance strategy is an essential goal with a necessity to reduce maintenance costs while increasing the device's useful life [11]. Due to myriad number of medical devices in healthcare facilities, plenty of methodologies has been adopted to ensure the devices are reliable. Prioritizing maintenance strategies is proposed to ensure uptime, budget utilization, reduce maintenance, and replacement cost by categorizing the devices to their criticality [12, 13]. Zamzam et al. [13] proposed three preventive and corrective maintenance models and replacement prioritization for comprehensive maintenance planning. A budget curtailment for maintenance expenses and replacement is essential and always a significant concern at the top management level. A prioritization technique to prevent failures is proposed for complex medical device systems and components, which may imply the patients directly [2]. Coherent medical device maintenance can ensure a longer lifespan, functionality, reliability and relies explicitly on the scientific and engineering principle, the application of biomedical engineering education, the previous maintenance history and experience, a recommendation from manufacturers, a suggestion from experts, and the obligation to comply to country regulatory requirements [14, 15]. Maintenance program's effectuality and efficiency are measured and evaluated through maintenance history data, physical inspection, and failure analysis using several techniques to minimize or mitigate the risks. The maintenance program should not be limited to the cost of ownership, maintenance cost per acquisition, clinical engineering costs for a group, cost of clinical engineering per occupied beds, cost of clinical engineering per patient discharged, and service contracts as a percentage of total maintenance and management costs [14].

The expertise level of users and biomedical staff is another factor that affects the reliability and failures of medical devices. Employing a skilled worker with expertise in biomedical engineering is a challenge, and maintenance contracting or outsourcing services is an alternative to overcome this limitation [2]. Malaysia had adopted a maintenance service contract for medical devices and implemented a privatization policy with Concession Company since 1997 due to the same constraint. Besides the importance of scheduled maintenance and competencies to ensure reliability, other factors influencing medical devices' performance are calibration work and electrical safety testing. Calibration is accomplished to ensure accuracy, and an electrical safety test is conducted to ensure the patient is safe from electrical leakage or injury. An investigation test conducted on six high-risk medical devices concluded that 58% of devices failed the performance test, higher than previous studies with 21% and 26%, respectively [16]. Moreover, approximately 9% of infusion pumps and 12.6% of dialysis machines do not meet electrical safety requirements when measurements are conducted at healthcare facilities [17, 18]. In Malaysia, an electrical safety test is imperative throughout schedule maintenance or a newly purchased medical device, and calibration work is committed as the manufacturer suggests on selected types of medical devices.

**Figure 1:**
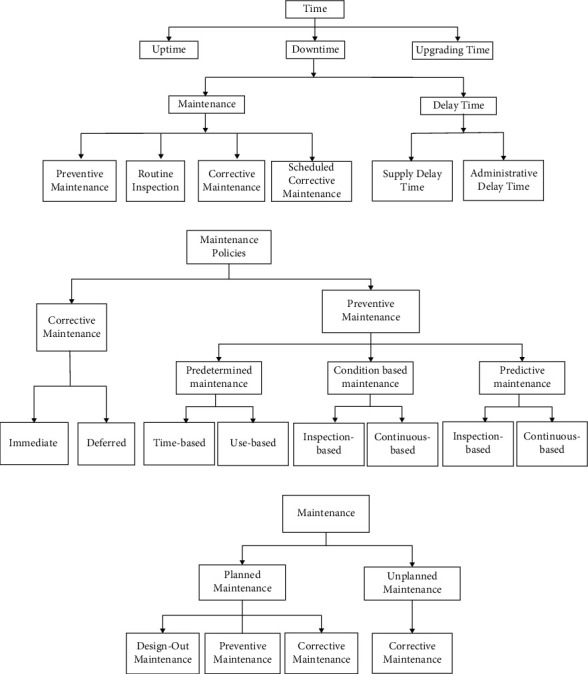
Medical devices maintenance category. (a) Malaysian government hospital practice with maintenance during downtime includes preventive maintenance, routine inspection, corrective maintenance, and scheduled corrective maintenance [15]. (b) Evolution through time for maintenance policies and categorizes maintenance to corrective and preventive maintenance [24]. (c) Planned maintenance is divided as design-out, preventive, and corrective maintenance correspondingly [21].

**Figure 2:**
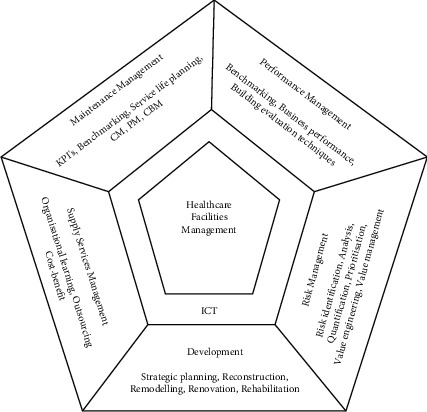
Six healthcare facilities management core domains represents five respective domains specifically maintenance management, performance management, risk management, development, and supply services management [23].

Maintenance for medical devices at Government Hospitals in Malaysia can be categorized into three primary classifications: uptime, downtime, and upgrading time with four types of maintenance, specifically preventive maintenance, routine inspection, corrective maintenance, and schedule corrective maintenance, as indicated in Figure 1(a). Similarly, in Figure 1(b), predetermined maintenance, condition-based maintenance, and predictive maintenance are used to explain the classification of maintenance. As the manufacturer suggests, preventive maintenance is accomplished in a specific time or interval throughout the year. The task is executed before failures occur or a planned replacement of wearable parts to prevent predicted failure before respective mean time to failures (MTTF) [19–21]. Routine inspection is a physical inspection of selected medical devices with minimal intervention. Meanwhile, corrective maintenance is performed subsequent to breakdown or fitting the existing errors [19]. In contrast, scheduled corrective maintenance is performed due to the extended time required to resolve the equipment breakdown issues that had been encountered during preventive maintenance [22]. Predictive maintenance is another proactive action applied to forecast the device's malfunctions by gathering data and predicting the errors that may occur in the future, decreasing the failure rate, and improving device utilization [19].  Figure 1(c)) in [21] uses an additional term: design out maintenance, construction, installation, start-up, and tuning in adopting maintenance program with similar interpretation. Besides, the medical devices management system is also embarked in the healthcare system, incorporating an equipment inventory, schedules for preventive maintenance, a work order system, outsourcing of contract management, and asset management [8]. Shohet and Lavy [23] proposed a framework on six interrelated healthcare facilities management (HFM) core domains to ensure an efficient management system. The core domains are illustrated in Figure 2 in the pentagon, with five sections reflecting one topic in HFM. There are two main sections from these core domains: Maintenance Management and Risk Management which will be elaborated further in the results and discussion section.

Based on recent global scenarios as illustrated before, although numerous studies have been conducted on medical device reliability, there is still a scarcity of review papers summarizing the bigger picture of problems associated with medical device reliability assessment. The results from systematic reviews are advantageous in identifying the gap and improving the current scientific knowledge. This study summarizes the last ten years' publications, and research gap has been identified for future work and improvement. This manuscript evaluates and execute a systematic review using the PRISMA technique to summarize, identify existing related studies in the same research area, analyses the significant parameters affecting medical device reliability, and appropriate techniques applied formerly to assess medical device condition and its reliability.

## 2. Materials and Methods

### 2.1. Identification of Reporting Guidelines Using PRISMA Technique

The review of various articles is adopted from PRISMA, and this technique has been established since 2009 and is widely used in healthcare facilities and clinical applications [25, 26]. The process involves searching specific keywords, selecting articles, screening, sorting inclusion and exclusion criteria, extracting data, and synthesizing. A systematic review on medical devices reliability is accomplished by searching in eight databases, namely Science Direct, Scopus, IEEE Explore, Web of Science, Emerald, MEDLINE Complete, Dimensions, and Springer Link, from 2010 to May 2021. The Google Scholar database was excluded from this review due to a high number of duplicate articles accessible by the reader.

### 2.2. Selection of Relevant Articles

The selection of relevant articles is achieved by using the inclusion and exclusion criteria as tabulated in Table 1. There are huge numbers of articles under the similar search string keywords extracted from various sources. The article is screened, and only relevant articles are selected. Keywords in Table 2 are applied with the medical device as the main keyword, and reliability, prioritization, maintenance, and machine learning are other keywords included in the selection process. Data analysis is performed by extracting essential techniques, parameters, and deliverables are tabulated to effectively recapitulate the content of the selected articles and lead to identification of scientific research gap. The inclusion criteria and exclusion criteria are used to screen and filter relevant articles in the timeline of the year 2010 to May 2021. The criterion is listed in Table 1, where only research articles, review articles, conferences, and proceedings papers are included. The period is limited from 2010 to May 2021 to ensure only the latest articles are selected. The target is to review, screen, and summarize articles with comprehensive content specific to the desired research area and optimize the outcomes. The selection of papers is challenging and time-consuming. There are difficulties selecting only related articles in the field with innumerable medical device publications that emphasize clinical applications, diseases, and patient care.

### 2.3. VOSViewer Mapping and Visualization

VOSviewer is a software tool for creating maps based on network data and for visualizing and exploring maps. This software can generate maps based on network data for visualization purposes. The maps use a network as a set of items and links between the items, whereas the cluster is a set of items included in a map. The boxes in the maps represent the object of interest, whereas the link in the connection represents the relationships among two different items. The width of the line shows the strength of the link, whereas a wider length indicates a higher number of publications are obtainable online. The cluster is segregated by different colors, which shows the set of items on the map [27]. This mapping aims to visualize existing literature and their relationship, which helps to view current topics available under medical device reliability and ensures these topics are included in this review.

**Figure 3:**
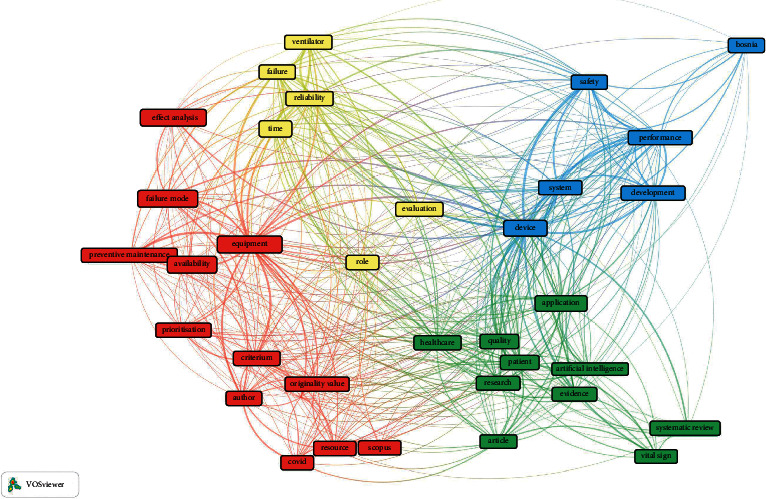
Overall overview of medical devices reliability with its relationship and linkage one another.

**Figure 4:**
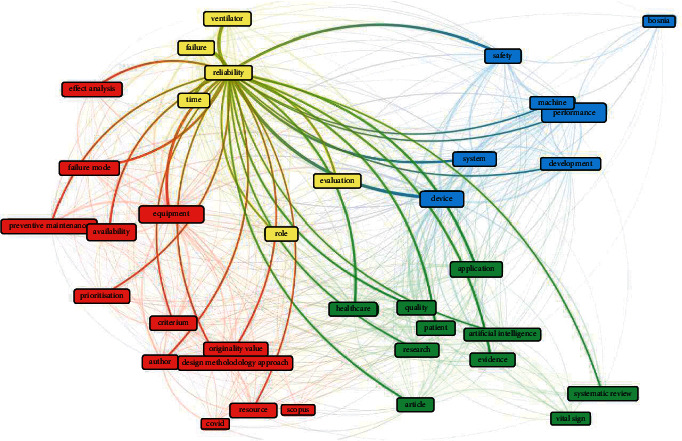
The relationship between reliability to main areas in other clusters.

Figure 3 illustrates an overall view of four different relationship clusters in medical device reliability studies where the most significant cluster or the strongest link is denoted in “red.” Four distinct clusters are differentiated with colors where “red” color represents 12 topics in the study, “green” represents ten topics, “blue” consists of six topics, and the “yellow” color is the smallest cluster with six topics. The main topics used in the largest cluster in Figure 3 are prioritization, preventive maintenance, failure mode, effect analysis, resource, availability, etc. Therefore, this study shall review these important topics by using all these keywords as a search string. The network is analyzed deeper by focusing only on reliability studies in Figure 4. This figure demonstrates a strong relationship to other clusters with a total of 36 items and is divided into four different clusters for reliability study. Cluster 1 in “red” color consists of 13 items: availability, effect analysis, equipment, failure mode, preventive maintenance, prioritization, resource, etc. Cluster 2 in the “green” color indicates 10 items, including artificial intelligence, healthcare, patient, quality, research, etc. The subsequent group of clusters namely Cluster 3 denoted in “blue” with seven different items comprises of development, device, machine, performance, safety, and system. Cluster 4 in “yellow” represents six items namely failure, reliability, ventilator, etc. These findings conclude that the topic mapped under the medical device reliability is huge, and the foremost topics are performance, artificial intelligence, failure, safety, etc. The result section will discuss further all these significant aspects on numerous topics available and their implication to medical device reliability for comprehensive understanding.

## 3. PRISMA Flowchart

**Figure 5:**
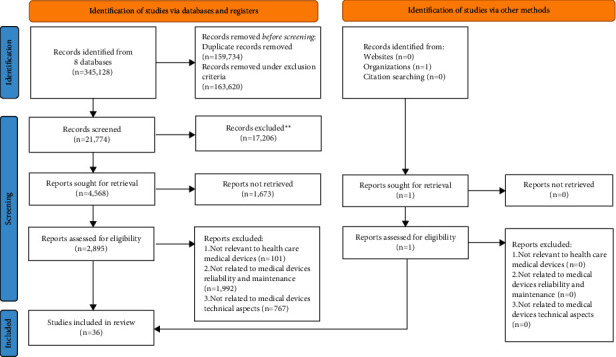
PRISMA framework for identification, screening, and inclusion process from 345,128 to 36 selected articles.

By adopting the PRISMA technique, there are 345,128 articles selected primarily from eight databases based on keywords obtained from VOS viewer network. The total number of articles is enormous due to a vast number of duplicate articles, and further screening is required to exclude the articles in the exclusion criteria. The Google Scholar database is excluded from this article screening to reduce the number of duplicated articles. The existing literature concludes 95% of Web of Science articles and 92% of Scopus articles were also found in Google Scholar [28]. After removing the duplicates, only 36 articles were screened thoroughly and included under this review, as indicated in Table 2. Meanwhile, Figure 5 shows the flowchart or process flow for article selection and screening based on the two main elements: identifying studies via databases and register and identifying studies via other methods from Government Contract Document for Privatization of Hospital Support Services at Government Hospitals in Malaysia.

## 4. Results and Discussion

### 4.1. Main Findings and Research Gap

**Figure 6:**
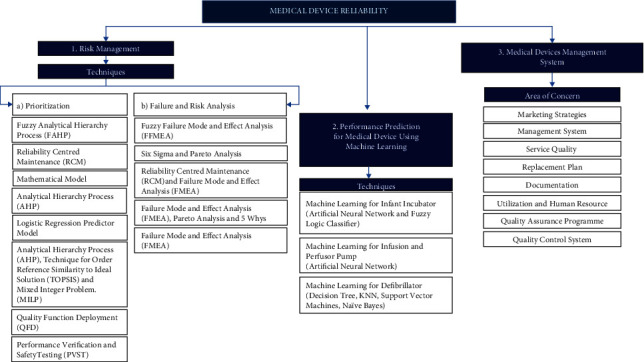
Summary on methodology applied for 36 selected articles. Three main areas are risk management (prioritization and failure and risk analysis), performance prediction for medical device using machine learning and medical devices management system.

The PRISMA framework and approach resulted in three primary areas under medical devices reliability: risk management, performance prediction for medical devices using machine learning, and medical device management system, as described in Figure 6. These three main topics are extracted from existing literature abridged in table form and summarized in Table 3. The research gap or future work was also extracted from the literature and tabulated in the table. Risk management is the most common topic used in wide applications, where prioritization, failure, and risk analysis are used. The standard techniques used in risk management topics are Failure Mode and Effect Analysis (FMEA), a combination of FMEA and Fuzzy (FFMEA), Analytical Hierarchy Process (AHP), etc.

Table 4 indicates the similarity in the methodology applied to assess risk management. From this result, AHP, FFMEA, FMEA, Mathematical Model, and Quality Function Deployment (QFD) have the most articles publications compared to the others. The same topics are conversed by Shohet and Lavy [23], where risk management and maintenance management is the two core domains in Six Healthcare Facilities Management, as deliberated in the Introduction section. The FFMEA technique is the latest combination technique applied in identifying the risk factors, mitigating failures, and contributing to medical device reliability of the devices. The main outcome in risk management is to determine or restructure the maintenance program, develop a maintenance strategy, suggest a maintenance interval, prioritize maintenance priority based on criticality, optimize budget expenditure, develop software, introduce a mathematical model, and propose a new comprehensive framework for maintenance purposes. Besides, the parameters affecting medical device reliability is highlighted such as equipment features, function, maintenance requirement, performance, risk and safety, failures, availability, utilization, and cost.

Meanwhile, performance prediction of medical devices using machine learning approach is another main topic where only three medical devices were involved in the study, specifically infant incubator [29, 30] infusion, and perfusion pump [31], and defibrillator [32]. However, the three medical devices are written in separate articles. This topic is subjected to more future work, where numerous medical devices are still available to be studied, especially on critical medical devices. Kovačević et al. [30] attained an accuracy of 98.5%, intending to forecast device functionality for infant incubators by two categories: accurate and faulty. A similar methodology is applied by Badnjevic et al. [33] for infant incubators and mechanical ventilators. A performance parameter value is utilized for a defibrillator and achieved excellent performance of 100% accuracy in Random Forest classifier to predict positive: for device passed inspection or negative: for faulty devices [32]. Meanwhile, Hrvat et al. [31] attained 98.06% accuracy based on conformity assessment, where the outcomes are identified as pass or fail for infusion and syringe pumps. These findings conclude although a model is developed in performance prediction study; however, the model does not apply to other types of medical devices, and there is missing of cost analysis impacts an existing maintenance program. A model with the ability to reduce the likelihood of failures can ensure the availability of services, especially during a pandemic.

Medical Devices Management System is another topic to ensure medical device reliability. Bahreini et al. [4, 8] distinguished the factors affecting medical equipment maintenance and management and are divided to resources, human resources, financial, physical documentation, education, service, quality control, information bank, inspection and preventive maintenance, training and education, management, services, design, and implementation. Medical Devices Management System in this review highlights marketing strategies [34], service quality [35, 36], replacement plan [37], utilization and human resource [10], quality assurance program [38], and quality control system [39], where the replacement plan, marketing strategies, and establishment of Standard Operating Procedure (SOP) are essential in management.

The research gap is identified with a future work column in Table 3, where previous medical device reliability focuses only on limited categories of medical devices under one research article. Most articles will include a certain number of medical devices depending on data available within a specific time period. Besides, a function parameter used in existing research has therapeutic, diagnostic, analytical, and miscellaneous categories. None of the article's highlights or focuses only on critical devices within the said categories. There is a limitation in gathering maintenance cost data, difficulties in accessing medical devices at healthcare facilities, limited years of medical devices in services, the proposed model do not apply to all types of medical devices, small sample size, limited number of hospitals involved, and there is no specific research is conducted globally on critical medical devices performance prediction using Artificial Intelligence (AI). The faulty critical medical device might lead to death and become an essential life-saving device in hospitals. When this study was written in May 2021, Malaysia is facing a third wave of COVID-19 pandemic and lockdown where most of hybrid COVID-19 hospitals running with a limited number of Intensive Care Unit (ICU) beds, limited number medical devices, especially ventilators, and relatively high bed occupancy rate (BOR). Similar impacts affect globally with the limitation of hospital beds and ventilators and involve making tough decisions in deciding patients to treat and patients to release to death [40]. Approximately one-third to one-half of patients in ICU require ventilator support in Wuhan, China, where the COVID-19 virus was first discovered in the world [41]. The shortage of critical medical devices in healthcare facilities implies service delivery to patients during this challenging time. Medical device reliability, functionality, maintenance, and other technical aspects are imperative to ensure patient service delivery. The study on performance prediction for medical devices using AI can be further explored with sufficient maintenance history data to train a model with the best accuracy. A predictive analysis model with the ability to predict impending failures can ensure the device's uptime, minimize future failures, and deliver better service to patients and the country, especially during this pandemic. Moreover, no similar study had been conducted in Malaysia from 2010 to 2021 on performance prediction for critical medical devices.

**Figure 7:**
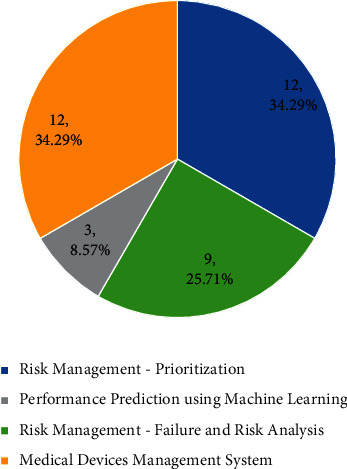
Percentage of main topics for 36 selected articles. The percentage for medical devices management system and prioritization (highest percentage with 34.29%), failure and risk analysis 25.71%, and performance prediction using machine learning (lowest percentage of 8.57%).

**Figure 8:**
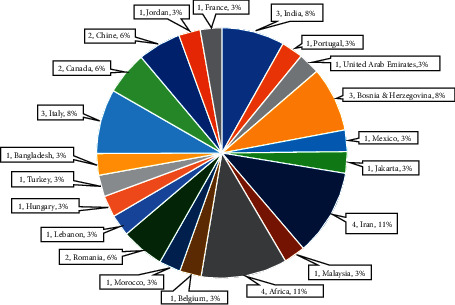
Percentage of 36 publications worldwide with the highest contribution at Iran and Africa (11%) followed by India, Bosnia Herzegovina, and Italy (8%). Most of the contribution is at 3% with only one publication available.

The 36 selected articles are analyzed through a pie chart percentage in Figure 7 to demonstrate the weightage between the three main topics described above. The highest knowledge contribution in 36 articles with 34.29% (12 publications) is on Medical Devices Management System and Prioritization, followed by 25.71% or equivalent to nine publications for Failure and Risk Analysis. The lowest percentage of current knowledge is on performance prediction for medical devices using AI or Machine Learning, contributing 8.57% with three publications from selected articles. Performance prediction can be improved by adding new scientific knowledge, data, or methodology to existing expertise and substantially concludes new or improved results. The geographic areas in Figure 8 showed that the medical devices reliability study in Iran and Africa denotes the highest percentage with four publications or 11%. India, Bosnia–Herzegovina, and Italy have three publications on this topic with an 8% contribution to existing knowledge. Contribution and awareness are still limited whereby only one article is published on this subject with a 3% percentage in most countries in the world.

### 4.2. Input Parameters in Medical Device Reliability Studies

The outcomes of medical device reliability studies had been discussed in the previous section with the diverse methodology applied to achieve the desired goals. The medical device input parameters are the pertinent element highlighted in the methodology to accomplish the outcomes.  Table 5 describes eight elements identified as the former methodology's main elements: features, function, performance, operation and maintenance, service availability, recall and hazards, cost, and utilization. The subcategory elements are listed based on outcomes achieved, and each parameter's application depends on the objectives. For example, age is an highly used parameter and is applied to five outcomes which are Prioritization, Prioritization for Preventive Maintenance, Performance Prediction, Replacement Plan, and Mathematical Model [12, 20, 32, 37, 42, 44, 45, 47, 52]. The explanation on parameters definition is explicated in the description column, and the outcomes column is organized in colors for better elucidation.

Table 6 summarizes the input parameters based on an application, where the highest demand for input parameters required is for prioritization for PM with 18 parameters distinctly. There are differences (in “bold”) between parameters applied for prioritization and prioritization for PM purposes. A future study using simulation results based on relevant datasets shall examine the most significant parameters to be used and select only significant parameters which influence the outcome instead of using all 18 parameters for prioritization purposes. Excessive selection of input parameters might change the accuracy of the result, and there is a probability of obtaining a different result by adding or removing parameters in the dataset. In machine learning or AI application, excessive predictors demand a higher training time during model development and are laborious.

## 5. Conclusions

The literature on medical device reliability is examined in this review, with 36 selected articles from 2010 to May 2021 being recapitulated, and research gaps are identified. PRISMA framework approach is used to investigate all articles in the medical devices' reliability study. The result concludes medical devices' reliability is categorized into three main areas: risk management, performance prediction using AI, and medical devices management system. Most studies emphasized prioritization, failure and risk analysis, and management systems. Performance prediction using AI shall be enhanced to reduce the likelihood of failures since only three papers are published under these areas, with only three medical devices involved. Medical devices are extensively used to treat COVID-19 patients in healthcare services and need to be operated without failure, especially during a pandemic A critical medical device is generally a life-saving device; high-end equipment with a high maintenance cost and uptime commitment is guaranteed for continuous service delivery. Future work shall highlight critical medical devices in a broader view and not limited to only selected medical devices. Code of Practice for Good Engineering Maintenance Management of Active Medical Devices in Malaysian Standard (MS2058) had listed 44 types of critical medical devices used at healthcare facilities [22]. The listed critical devices should be prioritized in reliability studies since most the devices are life-threatening devices in the therapeutic and diagnostic categories. They are mainly used in critical areas such as Operation Theatre (OT) and Intensive Care Unit (ICU) at healthcare facilities. Future work shall also examine the most significant parameters and select only substantial parameters that influence the overall result instead of choosing all the parameters from other authors' experiences. Based on all review articles discussed, new scientific data on action taken after failures, maintenance cost, larger dataset, and new predictive analysis model using AI are expected to improve existing knowledge with priority given only to critical medical devices.

## Figures and Tables

**Table 1:** Inclusion and exclusion criterion.

Criterion	Inclusion	Exclusion
Sources	Journal/research article	Chapter in book, book section, encyclopedia, magazines, early access, expert briefing, guidelines, and other sources
	Review article	
	Conference paper	
	Proceedings paper	
	Case study	

Language	English	Non-English

Period	Between 2010 and 2021	Less than 2000

Article selection	a) Related to the reliability, prioritization, maintenance, and machine learning	a) Not related to healthcare medical devices
		b) Not related to medical devices reliability and maintenance
		c) Not associated with medical devices technical aspects (clinical application, diseases, and patients care)

**Table 2:** Search strings for eight databases.

Searching texts	Science Direct	Scopus	Ieee Xplore	Web of Science	Emerald	Medline Complete	Dimensions	Springer Link
Prioritization and medical devices	8,477	123	21	69	285	3,165	3,278	1,837
Medical devices and maintenance	39,115	1,748	251	611	1,000	7,263	42,780	21,589
Medical devices and reliability	39,044	3,060	1,169	1,429	2,000	4,504	73,830	26,082
Medical devices and machine learning	15,443	234	90	1,897	1,000	0	14,300	29,434
Total including duplicates	102,079	5,165	1,531	4,006	4,285	14,932	134,188	78,942
Subtotal including duplicates	345,128							
Total selected articles	36							

**Table 3:** Existing literature on medical devices reliability and research gap.

Topic	Technique/area of concern	Geographic area	Medical device	Articles/year	Outcomes	Research gap/future work
Risk management (prioritization)	Fuzzy AHP	Portugal	Five selected medical devices	[42]/2020	Priority for renewing medical device categorized to low, medium, high and urgent for replacement	Only selected medical devices are included, and the most significant criteria are not specified.
						Limited no. of age in service
	RCM	Malaysia	Medical device	[43]/2019	Breakdown factors are divided into three which is maintenance services type, environmental and human factors	RCM method is widely used but requires sufficient data to complete the process
	Mathematical model	Mexico	16 selected medical devices	[44]/2020	The result determines the annual number and priority for PM. Type of equipment (highest priority), location (lowest priority)	The model applies to selected 16 medical devices
		South Africa	Infusion pump and ventilator	[20]/2013	The findings conclude age does not affect the survival of equipment due to the limited number of 5 years in service	Limited data for ventilators and only five years in age.
						Model not possible to be analysed to other devices due to insufficient data
		United States, France	Medical device	[14]/2010	Biomedical equipment is classified into the high, medium, and low category	Current maintenance strategies are effective but lack the evidence of being efficient
	AHP	Jakarta	Medical device	[12]/2019	The highest maintenance priority is an excimer laser, followed by a retinal laser and others	The most significant parameters which influence the result is not specified
		Canada	Medical device	[45]/2011	Maintenance is prioritized with scores. Higher score for high priority in maintenance management program	A higher score requires further investigation
	Logistic regression predictor model	Romania	Three selected medical devices	[46]/2017	Maintenance intervals are prioritized and developed based on risk group from low to high	No standard exists for assessing risk, and the tool uses current practices to establish a baseline
	AHP, TOPSIS, and MILP	Tunisia, Africa	Medical device	[47]/2017	The maintenance strategies framework is developed and divided into time-based, condition-based and corrective maintenance	The framework shall be enhanced to a mathematical model
	PVST	Istanbul, Turkey	16 selected medical devices	[6]/2016	The preventive maintenance schedule is developed for older technology and predictive maintenance for newer technology	A future study is required to investigate failures of other medical devices excluded from this study.
	QFD	Italy	Four selected medical devices	[48],	A comprehensive framework for PM priority is developed. The most important criteria are function, maintenance requirement, and others	Data was collected in 2012. The framework is tested only once during scheduled PM.
				[49]/2015		
Risk management (failure and risk analysis)	FFMEA	India	Ventilator	[50]/2020	Nine failure modes for ventilators are ranked based on the risk criteria from remote, low to very high	Application of fuzzy FMEA limited to ventilator
		Budapest, Hungary	Medical device	[51]/2016	Comparison between FMEA and FFMEA technique concludes FMEA is more accurate by involving different expert impacts weights	Difficulties in collecting each expert opinion for each risk as to the number of risks increases
		Canada	Five selected medical devices	[52]/2015	Framework for prioritization and budget allocation for maintenance are developed. A maintenance strategy is proposed from low to very high priority	Development of a risk-based maintenance software based on the suggested comprehensive framework
	Six sigma, Pareto analysis	India	Ventilator	[53]/2020	Common failures are identified, which are flow sensor, expiratory valve, calibration, battery, display and oxygen sensor	Automated real-time proactive RCA shall be enhanced to prevent failures
	RCM and FMEA	United Arab Emirates	Four selected medical devices	[54]/2020	Results examine the relationship between the current practice of PM, failure mode, and RCM action is identified based on failure modes	Failure data for only 1 year.
						Lack of maintenance cost data and limitation to run RCM pilot project in the hospital
	FMEA, Pareto analysis and 5 whys	Kenya, Belgium	Three selected medical devices	[55]/2018	Daily, weekly, monthly tasks and maintenance protocols are developed based on the failures identified	Only focuses on cobalt-60 radiotherapy machines and limitations to evaluate the effectiveness of proposed strategies on operation and maintenance
	FMEA	Italy	Medical device	[56]/2015	One leading company in the development of medical devices is selected, and the process/risk connected to the design of new devices are evaluated	Technique shall be applied to other leading companies and develop a more manageable approach to overcome FMEA limitation
		Sierra Leonne, Africa	Anesthesia machine	[57]/2014	Five failures mode are identified, which are resource availability, environmental, staff knowledge and attitudes, workload, and staffing	The sample size is small, with only two hospitals involved, and it is not convincing whether the findings are applicable to other settings
		China	Medical device	[58]/2014	Potential failures in human reliability of medical devices are evaluated with numerical values of risk factors	Propose to apply the model to different medical device design

Performance prediction for medical device using machine learning	Machine learning (artificial neural network and fuzzy logic classifier)	Sarajevo, Bosnia, and Herzegovina	Infant incubator	[29]/2020	Performance is predicted, and decision tree has the best properties compared to the other four algorithms with 98.5% accuracy based on performance output error	Model is applicable for infant incubators only with two years dataset period
	Machine learning (artificial neural network)	Sarajevo, Bosnia, and Herzegovina	Infusion and perfusor pumps	[31]/2020	Feedforward neural network with ten neurons in a single hidden layer has an accuracy of 98.06% for perfusion pumps, 98.83% for infusion pumps, and 98.41% for both	Research shall be extended by introducing new parameters such as maintenance history and spare parts replacement to enhance accuracy
Performance prediction for medical device using machine learning–cont'd	Machine learning	Bosnia and Herzegovina	Defibrillator	[32]/2019	Performance is predicted, and random forest has the best properties compared to the other four algorithms with 100% accuracy	Model is applicable for defibrillator only with three years dataset period

Medical devices management system (MDMS) (marketing strategies)	AHP (questionnaire)	Iran	Medical device	[34]/2019	Research on marketing strategies concludes most essential barriers are a managerial and strategic barrier	Fuzzy AHP technique shall be applied to examine the compatibility with human verbal and vague expressions

MDMS (management system)	Qualitative approach (interview)	Iran	Medical device	[4]/2019	Factors influencing medical device management systems are categorized into seven themes (resources preventive maintenance, design, implementation, etc.), with 19 subthemes	The themes subjected for further research in Iran or other countries to improve quality

MDMS (service quality)	AHP	Iran	Medical device	[36]/2019	4 Iranian public hospitals are ranked based on four criteria to evaluate hospital service quality	More hospital selection would provide a better benchmark

MDMS (service quality)	Qualitative approach (questionnaire)	Ghana, West Africa	Medical device	[35]/2019	Adequacy of healthcare resources is the most decisive factor compared to the other four service quality factors on patient satisfaction	Shall be enhanced to the district or regional hospital instead of a teaching hospital

MDMS (management system)	Literature review	Iran	Medical device	[8]/2018	Eighty-nine factors affected medical equipment maintenance management: Resources, education, service, quality, inspection, etc.	Some factors overlapped with each other

MDMS (maintenance strategies)	MCDM	Morocco	Medical device	[2]/2018	Maintenance strategies conclude risk (29%) as an essential criterion, followed by equipment function (14%).	Data collection is lacking and investigating external contributors such as heavy use and misuse and environmental factors on hidden failure event

MDMS (replacement plan)	—	Lebanon	35 selected medical devices	[37]/2016	A replacement plan is proposed with ranked criteria and sub-criteria depending on the urgency	Integration between hardware and software using Internet shall be executed to generate data on lifespan

MDMS (documentation)	—	Bangladesh	Ventilator	[59]/2015	Risk factor reduction and standard operating procedure (SOP) is developed. Concludes lack of adequately educated, and trained clinical engineers to be solved	Service contract with vendors for maintenance shall be developed

MDMS (utilization and human resources)	Qualitative approach (questionnaire)	India	Diagnostic medical device	[10]/2015	23% of the devices are underutilised	The sample size was small and limited to diagnostic devices in the histopathology lab in 2012

MDMS (quality assurance)	—	Bucharest, Romania	Radiant warmer, and infusion pump	[38]/2013	Risk and score for both devices are addressed with five different criteria as guidance in managing quality assurance program	Limited to only two types of medical devices. Maintenance software in the database shall be developed
MDMS (management system/Software)	—	Jordan	Medical device	[60]/2012	Presents a software system (EQUI-MEDCOMP) using microsoft visual basic (version 6) designed to improve maintenance management	Parameters used in the dataset are limited to 5 factors; other factors shall also be considered

MDMS (quality control)	—	China	9 selected medical devices	[39]/2010	A six-dimension risk model is proposed, and a quality control system is established	Quality control shall be enhanced to more types of medical devices

**Table 4:** Similarity on methodology techniques applied in existing literature.

Techniques	Articles
AHP	[12, 34, 36, 45]
Fuzzy FMEA (FFMEA)	[50–52]
Mathematical model	[14, 20, 44]
FMEA	[56–58]
Quality function deployment (QFD)	[48, 49]

**Table 5:** Input parameters categories in medical device reliability studies.

Category	Subcategory or input parameter	Description	Outcomes	Articles
Features	Functionality or performance inspection	Device condition	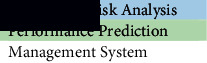	[50]
				[32]
				[60]
	Age	Device age	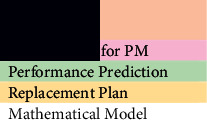	[42, 45, 47, 52]
				[12, 44]
				[32]
				[37]
				[20]
	Criticality	Device criticality	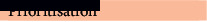	[45, 48, 49]
	Type of equipment	Device name	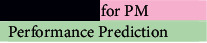	[44]
				[32]
	Class	Device classes	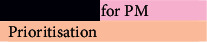	[12]
				[47]
	Manufacturer	Device supplier	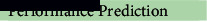	[32]

Function	Clinical function or intended function	Device function	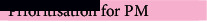	[12, 42]
			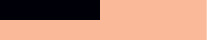	[44, 45, 47–49]
			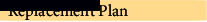	[37]
			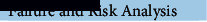	[38]

Performance	Error codes	Error on device	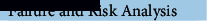	[50]
	Failures and frequency	Occurrence of failures	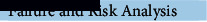	[50]
			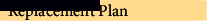	[37]
			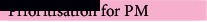	[12, 55]
			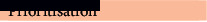	[48, 49]
	Root cause of failures	Root cause of failures	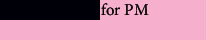	[55]
	Useful life ratio	The ratio between the age and expected life	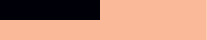	[48, 49]
	Service status of every failure mode	Status display on the device	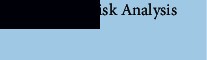	[50]
	Temperature error	Error on temperature	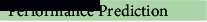	[29]
Operation and maintenance	Action taken/Repair notes	Tasks during repair work	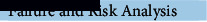	[50]
			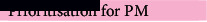	[55]
	Time to repair (downtime)	Time is taken during repair	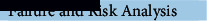	[50]
			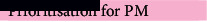	[12, 42, 55]
			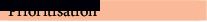	[47–49]
			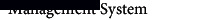	[60]
	Commission time	Time is taken for commission work	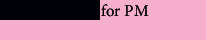	[55]
	Missed maintenance	Maintenance not to schedule	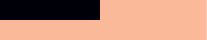	[48, 49]
	Parts or part usage	Spare parts inventory	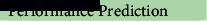	[29]
			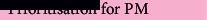	[42]
			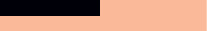	[35, 36]
	Maintenance interval	Frequency of maintenance	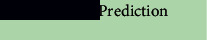	[46]
	Maintenance requirement or complexity	Maintenance activity based on device type	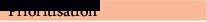	[31, 32]
			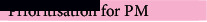	[2]
			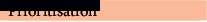	[34–36]
			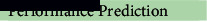	[48]
			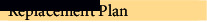	[52]
	Calibration	Calibration work	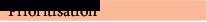	[31]

Service availability	Availability of support/alternative devices	Availability of backup device or outsource services	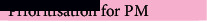	[2, 29]
			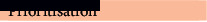	[32, 34]
			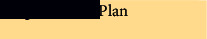	[52]
	Impact of operation	Operational impact	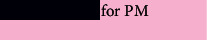	[29]
	Incident history	Incident record	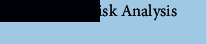	[54]
	No. of bed	Total bed	Management system	[55]
	Distance to nearest hospital	Measured distance	Management system	[55]
	Location	Device location	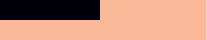	[31, 35, 36],
			Management system	[60]

Cost	Maintenance cost	Cost for spare parts and maintenance	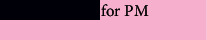	[12, 42]
Recall and hazards	Recall and hazards	Product recall and hazards	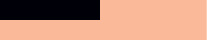	[44, 45, 47, 52]
			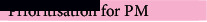	[12]
	Risk and safety	Risk and safety are associated with devices	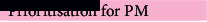	[12]
			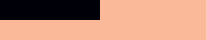	[45, 47–49]
			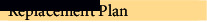	[37]
			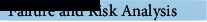	[38]
	Detectability	Ability to detect failures	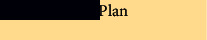	[37]

Utilization	Staff preferences	Staff preferences	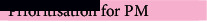	[42]
	Utilization coefficient/rate	Average total hours device is used	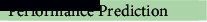	[29]
			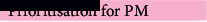	[12]
			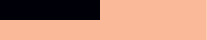	[45, 47–49, 52]
			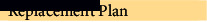	[37]

PM: Preventive Maintenance.

**Table 6:** Summary on input parameters based on outcomes.

Outcomes	Total parameters	Input parameters
Prioritization for PM	18	Age, **type of equipment**, **maintenance cost**, function, recalls and hazards, risk and safety, failures and frequency, utilization rate, **root cause of failures, staff preferences, action taken,** downtime, **commission time**, parts usage, maintenance requirement, availability of support/Alternative services, **impact of operation**
Prioritization	17	Age, **location, criticality,** availability of support/Alternative services, **class, distance to nearest hospitals,** function, failures, and frequency, **no. of bed, useful life ratio,** utilization rate, downtime, **missed maintenance**
		Parts usage, maintenance requirement, **calibration**, **functionality**, recalls, and hazards, risk, and safety
Failure and risk analysis	9	Functionality/Performance inspection, function, error codes, failure frequency, service status display on devices, action taken, downtime, incident history, risk and safety
Performance prediction	9	Functionality/Performance inspection, age, type of equipment, manufacturer, temperature error (depends on device), parts usage, maintenance interval, maintenance requirement, and utilization rate
Replacement plan	8	Age, function, failures and frequency, detectability, maintenance requirement, availability of support/alternative services, utilization rate, detectability, risk and safety

## Data Availability

The data used to support the findings of this study are available from the corresponding author upon request.
